# PKCε-CREB-Nrf2 signalling induces HO-1 in the vascular endothelium and enhances resistance to inflammation and apoptosis

**DOI:** 10.1093/cvr/cvv131

**Published:** 2015-04-16

**Authors:** Hayley Mylroie, Odile Dumont, Andrea Bauer, Clare C. Thornton, John Mackey, Damien Calay, Shahir S. Hamdulay, Joan R. Choo, Joseph J. Boyle, Allen M. Samarel, Anna M. Randi, Paul C. Evans, Justin C. Mason

**Affiliations:** 1 Vascular Sciences, Imperial Centre for Translational and Experimental Medicine, National Heart and Lung Institute, Imperial College London, Hammersmith Hospital, Du Cane Road, London W12 0NN, UK; 2 The Cardiovascular Institute, Loyola University Chicago Stritch School of Medicine, Maywood, IL, USA; 3 Department of Cardiovascular Sciences, University of Sheffield, Sheffield, UK

**Keywords:** Protein kinase C epsilon, Endothelium, Haem oxygenase-1, Inflammation, Apoptosis

## Abstract

**Aims:**

Vascular injury leading to endothelial dysfunction is a characteristic feature of chronic renal disease, diabetes mellitus, and systemic inflammatory conditions, and predisposes to apoptosis and atherogenesis. Thus, endothelial dysfunction represents a potential therapeutic target for atherosclerosis prevention. The observation that activity of either protein kinase C epsilon (PKCε) or haem oxygenase-1 (HO-1) enhances endothelial cell (EC) resistance to inflammation and apoptosis led us to test the hypothesis that HO-1 is a downstream target of PKCε.

**Methods and results:**

Expression of constitutively active PKCε in human EC significantly increased HO-1 mRNA and protein, whereas conversely aortas or cardiac EC from PKCε-deficient mice exhibited reduced HO-1 when compared with wild-type littermates. Angiotensin II activated PKCε and induced HO-1 via a PKCε-dependent pathway. PKCε activation significantly attenuated TNFα-induced intercellular adhesion molecule-1, and increased resistance to serum starvation-induced apoptosis. These responses were reversed by the HO antagonist zinc protoporphyrin IX. Phosphokinase antibody array analysis identified CREB1^(Ser133)^ phosphorylation as a PKCε signalling intermediary, and cAMP response element-binding protein 1 (CREB1) siRNA abrogated PKCε-induced HO-1 up-regulation. Likewise, nuclear factor (erythroid-derived 2)-like 2 (Nrf2) was identified as a PKCε target using nuclear translocation and DNA-binding assays, and Nrf2 siRNA prevented PKCε-mediated HO-1 induction. Moreover, depletion of CREB1 inhibited PKCε-induced Nrf2 DNA binding, suggestive of transcriptional co-operation between CREB1 and Nrf2.

**Conclusions:**

PKCε activity in the vascular endothelium regulates HO-1 via a pathway requiring CREB1 and Nrf2. Given the potent protective actions of HO-1, we propose that this mechanism is an important contributor to the emerging role of PKCε in the maintenance of endothelial homeostasis and resistance to injury.

## 1. Introduction

The vascular endothelium exhibits remarkable functional diversity and plasticity, both constitutively and in response to soluble, cellular, and physical factors. In addition to sustaining blood flow, the endothelium controls vascular permeability and angiogenesis, and maintains an anti-coagulant and anti-adhesive surface. Endothelial homeostasis is closely linked to well-being and its prolonged disruption is associated with a variety of diseases. Endothelial dysfunction, such as that seen in patients with diabetes mellitus, renal impairment, and systemic inflammatory diseases, is a critical early step in atherogenesis. Dysfunction reflects endothelial injury and indicates failure to perform homeostatic functions. Pathogenesis is multifactorial and includes excessive generation of reactive oxygen species that interact with and consume nitric oxide (NO), generating peroxynitrite and resulting in oxidation of tetrahydrobiopterin and promotion of NO uncoupling. Additional injurious factors include low-density lipoproteins modified by oxidation, pro-inflammatory cytokines, and complement activation. A vicious cycle of inflammation and further oxidative stress results in increased endothelial permeability to lipoproteins and monocytes, and ultimately to atherosclerotic plaque development.^1^ Although therapeutic reversal of endothelial dysfunction is clinically very attractive, achieving this is challenging and options remain limited.

Haem oxygenase-1 (HO-1) is the inducible form of haem oxygenase, and the rate-limiting factor in the catabolism of haem into biliverdin, releasing free iron and carbon monoxide.^2^ Biliverdin is converted to bilirubin by biliverdin reductase, while the increased intracellular iron induces expression of heavy chain ferritin, an iron-binding protein,^3^ along with the opening of Fe^2+^ export channels.^4^ Through their anti-inflammatory, anti-apoptotic, and anti-oxidant actions, the products of HO-1 enzyme activity play a central role in endothelial cytoprotective responses.^5^ This is well illustrated by the severe phenotype reported in human *HMOX1* deficiency, manifested by generalized inflammation, leucocytosis, haemolytic anaemia, and tissue iron deposition. There was evidence of severe endothelial injury and dysfunction, associated with abnormalities of coagulation–fibrinolysis and accelerated atherosclerosis.^6,7^ Human and rodent experimental data also suggest that HO-1 activity is anti-atherogenic. Thus, crossing *Hmox1*^−/−^ mice with atherosclerosis prone *ApoE*^-/-^ animals resulted in more extensive and complex plaques,^8^ while HO-1 induction impeded atherogenesis and stabilized plaques.^9^

The diversity of its protective actions has led to considerable interest in therapeutic modulation of HO-1. However, optimal means for targeting HO-1 or delivering its products remain to be determined, and functional data demonstrating efficacy in man are very limited.^5^ The low basal activity of the enzyme, combined with its inducibility, offers a potential therapeutic approach, and hence the upstream signalling pathways and transcriptional regulation of HO-1 remain a subject of intense interest.^5,10^ Phosphoinositide 3 kinase (PI3K) and mitogen-activated protein kinase (MAPK) pathways have been implicated in HO-1 regulation.^10–12^ Extracellular signal-regulated kinase 1/2 (ERK1/2), p38, and c-Jun N-terminal kinase (JNK) MAPK may act as both positive and negative regulators of HO-1 in a cell type and agonist-specific manner.^10,13^

Protein kinase C is a family of phospholipid-dependent serine/threonine kinases, divided on the basis of structure and response to phosphatidylserine, calcium, and diacylglycerol, into classical (α, β, and γ), novel (δ, ε, η, and θ), and atypical (ζ, ι, and γ) isoforms. Unique cell-specific functions of individual isoforms have been described, reflecting differences within isoform structure, subcellular compartmentalization, and PKC–target protein interactions.^14^ We reported that activation of the novel isoform PKCε enhances the resistance of human endothelial cells (ECs) to pro-apoptotic and pro-inflammatory stimuli.^15,16^ This led us to investigate the hypothesis that HO-1 represents an important downstream effector mechanism for the cytoprotective actions of PKCε. The current study reveals that specific targeting of PKCε in the vascular endothelium activates a novel cAMP response element-binding protein (CREB)-nuclear factor (erythroid-derived 2)-like 2 (Nrf2)-dependent signalling pathway, which induces HO-1 to enhance protection against inflammation and apoptosis.

## 2. Methods

### 2.1 Reagents

Anti-HO-1 (Cambridge Biosciences Ltd, Cambridge, UK), anti-phospho-PKCδ^(Thr505)^, anti-CREB, anti-phospho-CREB^(Ser133)^ (Cell Signaling, Danvers, MA, USA), anti-phospho-PKCε^(Ser729)^ (Upstate Ltd, Dundee, UK), anti-PKCε, and anti-Nrf2 (Santa Cruz Biotechnology, Santa Cruz, CA, USA). TNF-α was from R&D Systems (Abingdon, UK); angiotensin II (Ang II) and zinc protoporphyrin IX (ZnPPIX) from Sigma-Aldrich (Poole, UK).

### 2.2 Human and murine cell culture

The use of human EC conformed to the principles outlined in the Declaration of Helsinki, and was approved by the Hammersmith Hospitals Research Ethics Committee (ref no. 06/Q0406/21). Following informed consent, human umbilical vein ECs (HUVECs) were isolated and cultured as described.^17^ To generate matched wild-type (WT) and PKCε^−/−^ EC lines, PKCε-deficient mice^18^ (gift from Prof. P. Parker, Kings College London) were backcrossed on to a C57BL/6 background, and then crossed with H-2K^b^-tsA58 transgenic mice and the offspring crossed to generate H-2K^b^-tsA58/PKCε^−/−^mice. The H-2Kb-tsA58 transgenic mice (Immortomouse) were bred in house. The inducible tsA58 TAg allows murine cardiac ECs (MCECs) cells to be rapidly expanded under permissive conditions (33°C), before switching to 37°C for experimentation.^19,20^ Mice were housed under controlled climactic conditions in microisolator cages with autoclaved bedding. Irradiated food and drinking water were freely available. All animals were studied according to the guidelines from Directive 2010/63/EU of the European Parliament, with ethical approval from Imperial College London under UK Home Office Licence number PPL 70/6722. Prior to study, mice were sacrificed by CO_2_ inhalation followed by cervical dislocation. MCECs were obtained from six female PKCε^−/−^ mice and six PKCε^+/+^ littermate controls, using enzymatic digestion of hearts and positive selection with anti-endoglin and anti-Ig coated microbeads.^20^ EC phenotype was validated by flow cytometry, which demonstrated >95% positive staining with *Griffonia Simplicifolia Lectin I* (Vector Laboratories, Peterborough, UK) and anti-endoglin mAb MJ7/18 (Developmental Studies Hybridoma Bank, University of Iowa, Iowa City, IA, USA).

### 2.3 Adenoviral infection

The constitutively active (CA)-PKCε, CA-PKCδ, dominant-negative (DN)-PKCε, WT-PKCε, and Ad0 control adenoviruses were amplified in human embryonic kidney 293A cells, purified, and titrated as described.^21,22^ HUVECs were infected by incubation with adenovirus in serum-free M199 for 2 h at 37°C. The medium was replaced with M199 containing 10% fetal bovine serum (FBS) and 7.5 μg/mL of EC growth factor (Sigma). Optimal multiplicity of infection (MOI) for the CA-PKCε adenovirus, expressed as infectious units (ifu) per cell, was previously determined by immunoblotting.^15^

### 2.4 siRNA transfection

GeneFECTOR (3 : 50; VennNova, Parkland, FL, USA) and siRNA (40 nM final) were diluted separately in Opti-MEM I (Invitrogen, Paisley, UK). Equal volumes of siRNA and GeneFECTOR solutions were mixed and incubated at r/t for 5 min. Transfection solutions were added to HUVECs cultured in Opti-MEM I medium. After incubation for 4 h, culture medium was replaced with EGM2 medium (Lonza, Wokingham, UK) overnight and then with M199/10% FBS. CREB1 target sequence 1: 5′-AACCAAGTTGTTGTTCAAGCT-3′ (Qiagen Ltd, Sussex, UK). siGENOME SMART pooled oligonucleotides and the siGENOME control non-targeting siRNA #1 were from Thermo Scientific Fisher, Waltham, MA, USA.

CREB1 pooled sequences:

- 5′-GAGAGAGGTCCGTCTAATG-3′;
- 5′-CGTACAAACATACCAGATT-3′;
- 5′-GAGTGGAGATGCAGCTGTA-3′;
- 5′-TGACTTATCTTCTGATGCA-3′;

Nrf2 pooled sequences:

- 5′-TGACAGAAGTTGACAATTA-3′
- 5′-TAAAGTGGCTGCTCAGAAT-3′
- 5′-CCAAAGAGCAGTTCAATGA-3′
- 5′-GAGAAAGAATTGCCTGTAA-3′

### 2.5 Quantitative real-time PCR

Quantitative real-time PCR (qRT-PCR) was performed using an iCycler (BioRad, Hercules, CA, USA) and data calculated in relation to the β-actin and glyceraldehyde-3-phosphate dehydrogenase housekeeping genes. DNase-1-digested total RNA (1 µg) was reverse-transcribed using 1 µM oligo-dT and Superscript reverse transcriptase (Invitrogen). cDNA was amplified in a 25 µL reaction containing 5 µL of cDNA template diluted 1 : 30, 12.5 µL of SYBR supermix (BioRad), 5 pmol of sense and antisense gene-specific primers, and H_2_O to adjust the volume. The cycling parameters were 3 min at 95°C, 40 cycles at 95°C for 10 s, and 56°C for 45 s. Primer sequences: HO-1: forward 5′-TTCTATCACCCTCTGCCT-3′, reverse 5′-CCTCTTCACCTTCCCCAACA-3′. Nrf2: forward 5′-TACTCCCAGGTTGCCCACA-3′, reverse 5′-CATCTACAAACGGGAATGTCTGC-3′.

### 2.6 Immunoblotting

Aortas were snap frozen in liquid nitrogen, ground-up, and lysed in RIPA buffer containing a protein inhibitor cocktail, prior to gel-electrophoresis and transferred to a nitrocellulose membrane (Roche Diagnostics, UK). Immunoblotting of aortic and EC lysates was performed as described previously.^22^ To control for sample loading, membranes were re-probed with an α-tubulin Ab. Relative levels of protein expression were quantified using Lab-Works gel-pro (Ultra-Violet Products, Upland, CA, USA).

### 2.7 Phosphokinase antibody array

HUVECs were transfected with CA-PKCε or Ad0 adenoviruses and lysed after 16 h. Protein concentration was determined using a BioRad protein assay kit and 150 µg of protein lysate used for the human phosphokinase Ab array kit (R&D Systems), performed as per the manufacturer's instructions. Membranes were developed with horseradish peroxidase-conjugated streptavidin and visualized with a chemiluminescence substrate (GE-Healthcare Life Sciences, Little Chalfont, UK).

### 2.8 Flow cytometry

Flow cytometry was performed as previously described in detail.^17^ intercellular adhesion molecule-1 (ICAM-1) was detected with mAb 6.5B5 (generated in house) and FITC-rabbit-anti-mouse (Dako, Stockport, UK). The results are expressed as the relative fluorescent intensity, representing mean fluorescent intensity (MFI) with test mAb divided by the MFI using the secondary antibody alone.

### 2.9 Nrf2 activation assays

A TransAM Nrf2 ELISA kit (Active Motif, Carlsbad, CA, USA) was used to determine the transcriptional activation of Nrf2 as per the manufacturer's instructions. Nuclear extracts (5 μg) were added to a 96-well plate pre-coated with an anti-oxidant response element (ARE) consensus-binding oligonucleotide (5′-GTCACAGTGACTCAGCAGAATCTG-3′) and incubated for 1 h at r/t. A WT oligonucleotide was used as a binding competitor. Primary antibody (1 : 1000) was added for 1 h at r/t and specific binding was estimated by spectrophotometry, after incubation with a horseradish peroxidase-conjugated antibody.

Nrf2 nuclear translocation assays were performed with HUVECs cultured on gelatin-coated coverslips and fixed in methanol. ECs treated with 10% normal goat serum at 4°C overnight were treated with rabbit anti-Nrf2 or an isotype-matched control Ab, followed by goat anti-rabbit AlexaFluor 488 (Invitrogen) for 1 h, washing and staining with Draq5, and mounting in Vectashield (Vector). Nrf2 translocation was analysed by confocal microscopy, using images obtained at ×40 magnification from 10 fields of view per experiment, and quantified after correcting for autofluorescence and defining threshold intensity from background fluorescence, with results presented as MFI.

### 2.10 Cell survival assay

Analysis of EC survival was performed using the Promega (Southampton, UK) CellTiter96 [3-(4,5-dimethylthiazol-2-yl)-5-(3-carboxymethoxyphenyl)-2-(4-sulfophenyl)-2H-tetrazolium] (MTS) assay according to the manufacturer's instructions. The assay was quantified by recording absorbance at 490 nm. Percent cell death was calculated as follows: cell death = 100 − (OD test/OD control × 100), where control represents EC cultured in M199/20% FBS alone for the duration of the experiment.

### 2.11 Statistical analysis

Data were grouped according to treatment and analysed using the GraphPad Prism Software (San Diego, CA, USA). Numerical data are presented as the mean of individual experiments ± standard error (SEM). As indicated in the figure legends, differences between treatments were evaluated using either an unpaired Student's *t*-test or a one-sample *t*-test to compare two columns, while to evaluate three or more samples the one-way analysis of variance (ANOVA) was used. A Bonferroni correction was used to correct for multiple comparisons. A value of *P* < 0.05 was considered significant.

## 3. Results

### 3.1 PKCε regulates HO-1 expression in EC

**Figure 1:**
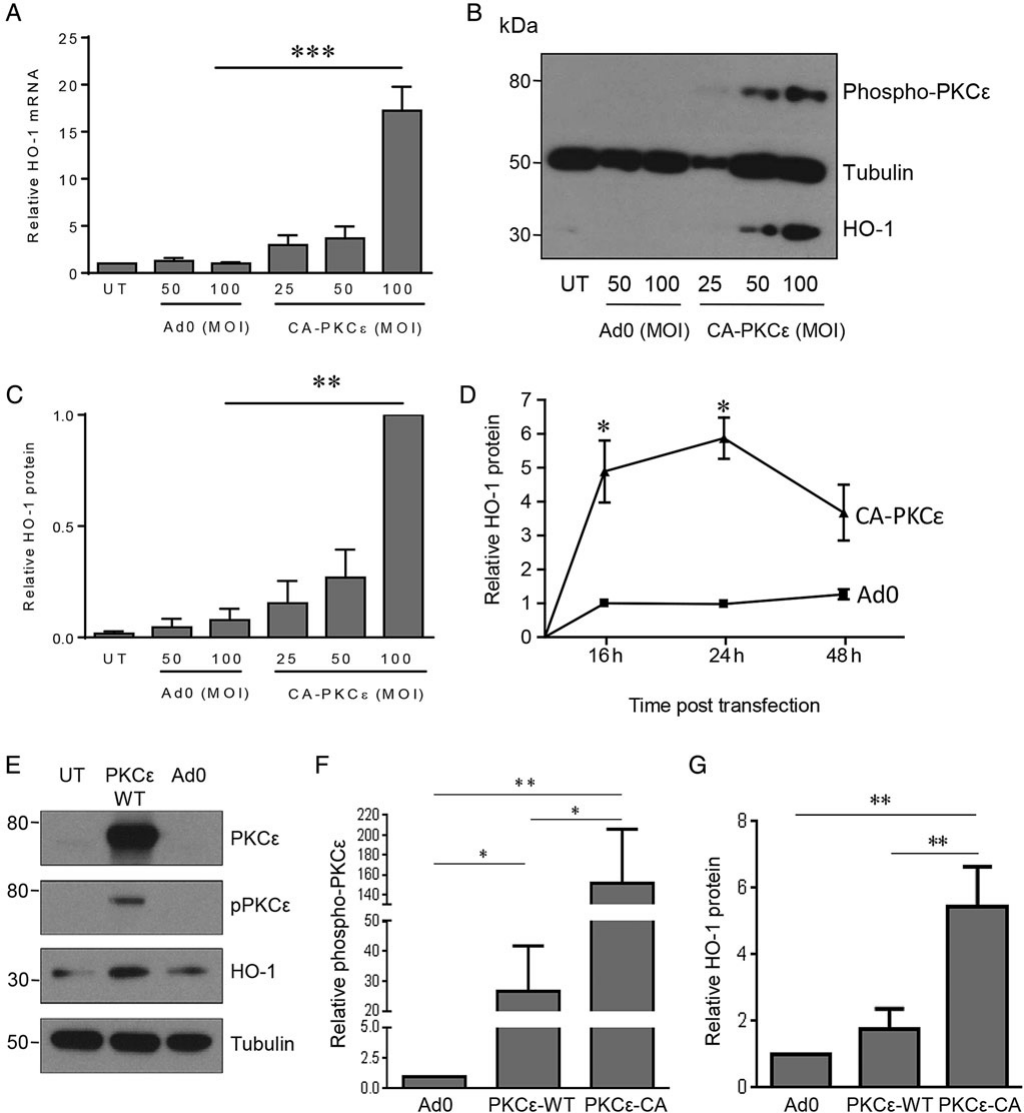
PKCε activation induces HO-1. HUVECs were left untreated (UT) or transfected with Ad0 or CA-PKCε-Adv for up to 24 h (MOI 0–100 ifu/cell), prior to lysis and quantification of (*A*) HO-1 mRNA by qRT-PCR at 16 h, or (*B*) HO-1 protein and phosphorylated PKCε^(Ser729)^ by immunoblotting after 24 h, with (*C*) a histogram showing pooled quantification data from five HO-1 immunoblot experiments. (*D*) HUVECs were left UT or transfected with Ad0 or CA-PKCε (MOI 100) for up to 48 h and HO-1 analysed by immunoblotting (*n* = 3). (*E*, *F*, and *G*) HUVECs were left UT or transfected with Ad0 or WT-PKCε-Adv for up to 24 h (MOI 25 ifu/cell), prior to lysis and quantification of PKCε, PKCε^(Ser729)^, and HO-1 by immunoblotting (*n* = 3). Data are presented as mean ± SEM normalized to the UT control except in (*C*), where densitometry data are presented relative to the maximal expression with CA-PKCε (MOI 100), on account of minimal basal HO-1 protein expression (ANOVA, *P < 0.05, **P < 0.01, ***P < 0.001).

PKCε is constitutively expressed in vascular EC and enhances resistance to injury.^15,16^ To explore underlying mechanisms, and specifically to investigate the hypothesis that the enzyme HO-1 contributes to the vasculoprotective actions of PKCε, we initially adopted a gain-of-function approach. Transfection of a well-characterized adenoviral vector (CA-PKCε-Adv), in which amino acids 154–163 were deleted in the inhibitory pseudosubstrate domain to render PKCε CA,^21^ resulted in a significant increase in HO-1 transcript levels when compared with a control adenovirus, maximal 16 h post-transfection of CA-PKCε (*Figure 1A*). This response corresponded to an increase of the activated phosphorylated form of PKCε^(Ser729)^ in HUVECs infected with the CA-PKCε-Adv, maximal at an MOI of 50–100 ifu/cell (*Figure 1B*).

**Figure 2:**
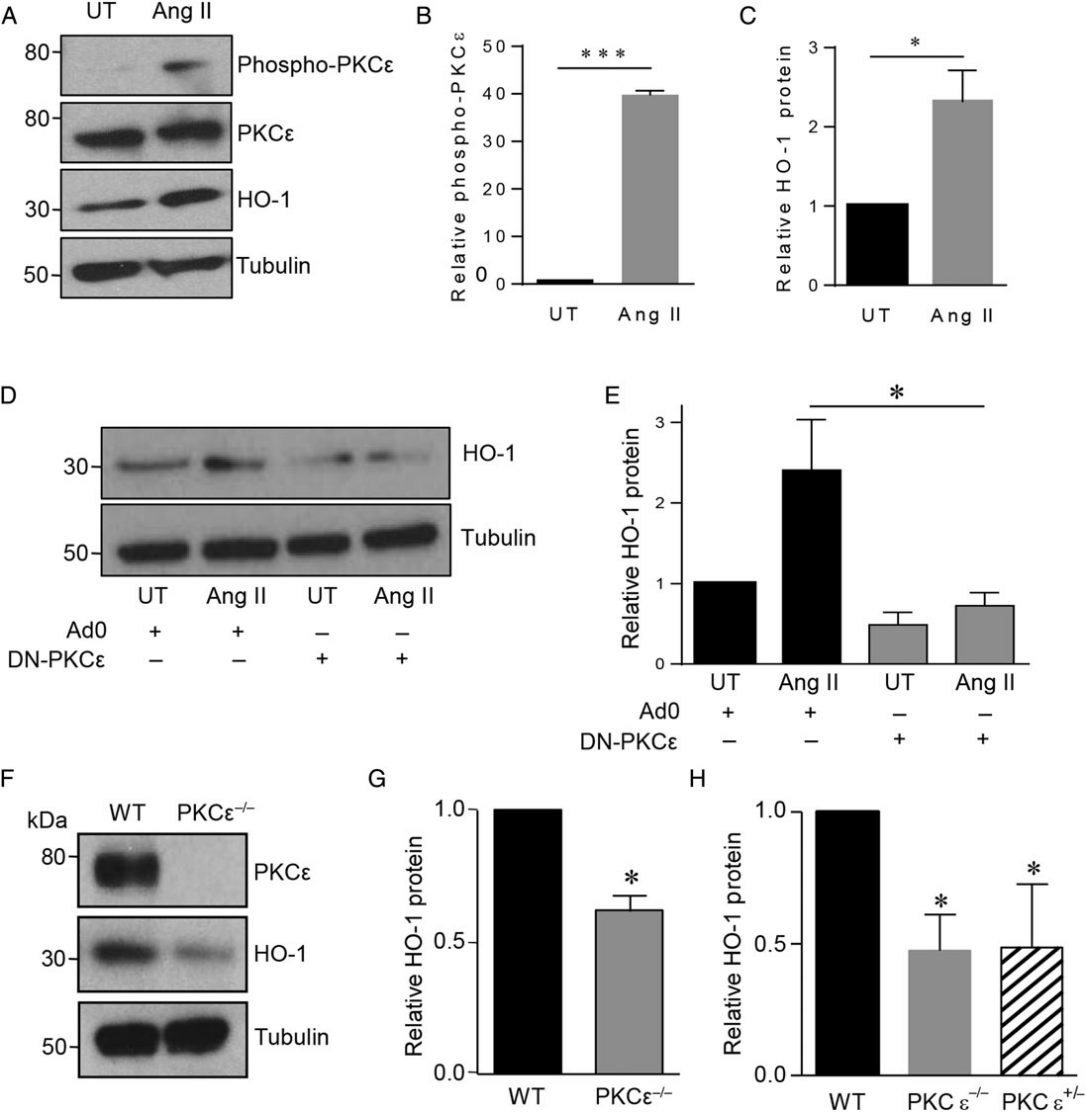
Effect of PKCε activation or depletion on HO-1. (*A*) HUVECs were treated with vehicle (UT) or angiotensin II (100 nM) (Ang II) for 16 h prior to analysis of PKCε, PKCε^(Ser729)^, and HO-1 by immunoblotting, and quantification by densitometry of: (*B*) PKCε phosphorylation and (*C*) HO-1 (*n* = 3). (*D*) HUVECs were infected with Ad0 or Ad-DN-PKCε (MOI 100) prior to treatment with vehicle (UT) or Ang II for 16 h and analysis of HO-1 induction by immunoblotting and (*E*) quantification by densitometry (*n* = 3). (*F*) Cardiac ECs from WT and PKCε^−/−^ mice were lysed and immunoblotted for PKCε, HO-1, and tubulin. (*G*) The histogram shows densitometric quantification of HO-1 (*n* = 6). (*H*) Aortas from adult PKCε^−/−^, PKCε^+/−^ mice, and WT littermate controls (*n* = 3) were harvested, snap frozen, and subsequently lysed and immunoblotted for HO-1 and tubulin. Data are presented as mean ± SEM, corrected for tubulin expression, and normalized to the untreated or Ad0 control (*A*–*D*) and to WT control (*G* and *H*) [one-sample *t*-test (*B*, *C*, *G*, and *H*), *t*-test (*E*), *P < 0.05, ***P < 0.001].

Although basal levels of HO-1 protein in untreated HUVECs were typically low or undetectable, a significant induction was seen 24 h following expression of CA-PKCε (*Figure 1B* and *C*). HO-1 up-regulation was maximal (up to six-fold) at 24 h, sustained at 48 h, and not seen in cells transfected with Ad0 (*Figure 1D*). In contrast to CA-PKCε, adenoviral expression of WT-PKCε led to a minimal increase in phosphorylated PKCε^(Ser729)^ and HO-1 protein (*Figure 1E*). Thus, induction of HO-1 is proportionate to the activation of PKCε (*Figure 1F* and *G*). The PKCε-mediated increase of HO-1 protein was completely attenuated by pretreatment with actinomycin D (not shown) and cycloheximide (see Supplementary material online, *Figure S1A*), confirming dependence upon gene transcription and *de novo* protein synthesis. To investigate physiological activation of this pathway, HUVECs were treated with Ang II, previously reported to activate PKCε.^23^ Ang II treatment resulted in a marked increase in phosphorylated PKCε^(Ser729)^, maximal after 15 min. Likewise, significant induction of HO-1 was seen after 16 h (*Figure 2A*–*C*). To determine the role of PKCε in HO-1 induction, ECs were infected with DN-PKCε-Adv or Ad0 prior to exposure to Ang II. PKCε inhibition reduced the low-level constitutive expression of HO-1 and also significantly attenuated the induction afforded by Ang II (*Figure 2D* and *E*).

Of note, PKCδ activity has also been linked to HO-1 induction in EC.^24^ To investigate a potential role for PKCδ, we first demonstrated that expression of CA-PKCε did not lead to phosphorylation of PKCδ (see Supplementary material online, *Figure S1B*). Although transfection of CA-PKCδ increased PKCδ phosphorylation at Thr^505^, it failed to induce HO-1 in HUVEC (see Supplementary material online, *Figure S1C* and *D*).

Next, PKCε-deficient mice were used to examine the effect of PKCε deletion on HO-1. The mice were bred on to a C57Bl/6 background for 10 generations prior to crossing with the H-2K^b^-tsA58 transgenic mice. This approach allowed the isolation of matched conditionally immortalized cardiac EC from H-2K^b^-tsA58/PKCε^−/−^ animals and WT littermates.^20^ HO-1 protein was detectable in WT EC, and expression was significantly reduced in cells derived from the PKCε-deficient mice (*Figure 2F* and *G*). Likewise, immunoblotting analysis of aortae harvested from C57Bl/6 PKCε^+/−^, PKCε^−/−^, and WT littermates revealed a significant reduction in HO-1 protein of up to 60% (*Figure 2H*). The depletion of HO-1 was equivalent in PKCε^+/−^ and PKCε^−/−^ mice, demonstrating the importance of PKCε, since loss of a single allele was significant.

### 3.2 PKCε-induced signalling pathways and HO-1

**Figure 3:**
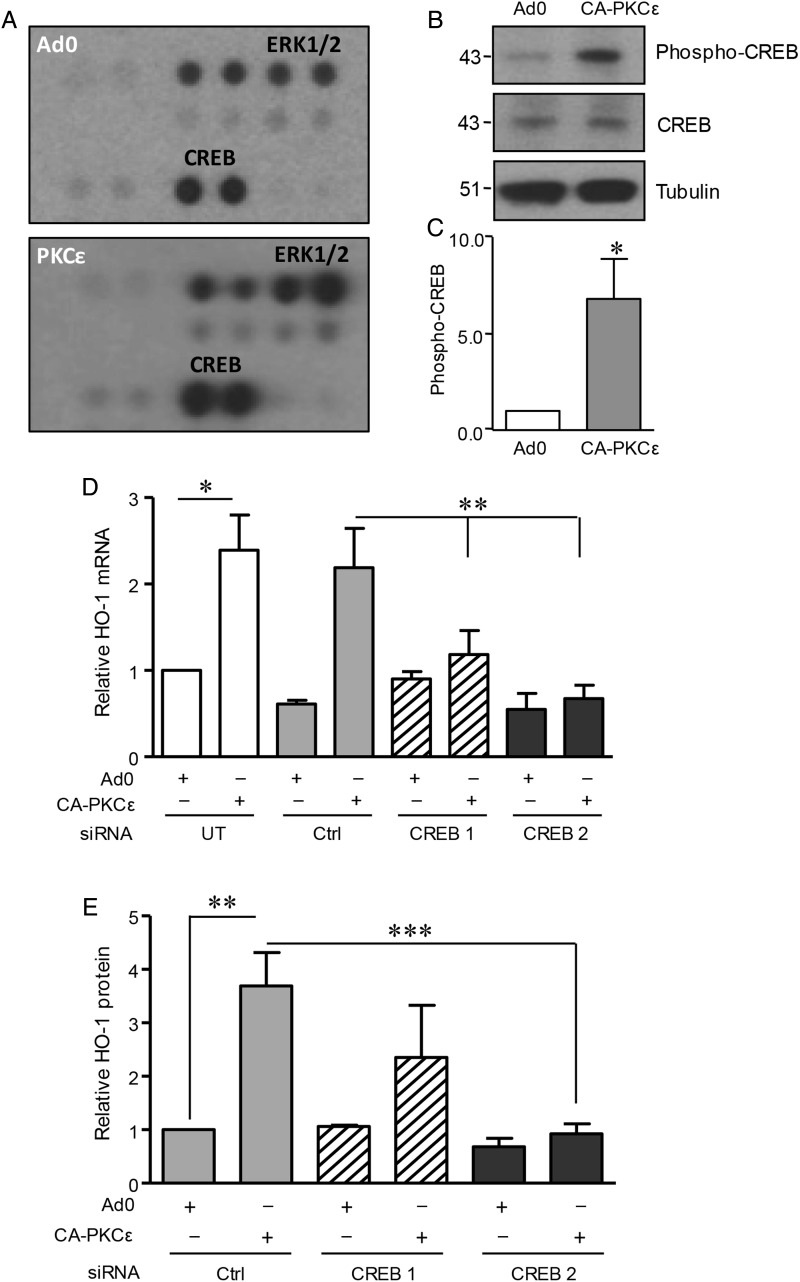
Role of CREB1 in PKCε-mediated HO-1 induction. (*A*) HUVECs were transfected with CA-PKCε or Ad0 (MOI 100) for 16 h prior to generation of lysates to probe a phosphokinase Ab array. Results for the phosphorylation of ERK1/2 and CREB1 are shown and are representative of two separate experiments. (*B*) HUVECs were transfected with CA-PKCε or Ad0 (MOI 100) for 16 h and immunoblotted for phospho-CREB^(Ser133)^, CREB1, and tubulin, with (*C*) a histogram showing pooled quantification data from five experiments. (*D*) HUVECs were left UT or transfected with a single CREB1 siRNA oligo (CREB1), pooled CREB1 siRNA oligos (CREB2), or control (Ctrl) siRNA (40 nM) for 24 h prior to transfection with control Ad0 or CA-PKCε adenovirus (MOI 100) for 16 h. HUVECs were lysed and HO-1 mRNA levels analysed by qRT-PCR. (*E*) HUVECs were transfected as above with Ctrl, CREB1, or pooled CREB1 siRNAs, prior to analysis of HO-1 by immunoblotting. The figure shows a representative blot. Pooled HO-1 quantification data from at least four individual experiments were corrected for tubulin expression where appropriate. Data are presented normalized to control-transfected cells (mean ± SEM). One-sample *t*-test (*C*), ANOVA (*D* and *E*), *P < 0.05, **P < 0.01, ***P < 0.001.

ERK1/2 and PI-3K/Akt signalling pathways may be activated by PKCε and have been implicated in HO-1 regulation.^12,15,16^ However, in human EC, neither MEK-1 inhibitor UO126 nor PI-3K antagonist LY290042 affected the induction of HO-1 mRNA (see Supplementary material online, *Figure S2A* and *B*) or protein (not shown) by CA-PKCε. This result led to experiments seeking alternative signalling intermediates, making use of a phosphokinase antibody array. HUVECs were transfected with CA-PKCε or Ad0 adenoviruses for 16 h and initially analysed by immunoblotting to confirm activation and phosphorylation of PKCε^(Ser729)^. These lysates were then used to probe the array. In addition to the expected increase in ERK1/2 phosphorylation in CA-PKCε-transfected cells, there was also marked phosphorylation of CREB1 at Ser^133^ (*Figure 3A*). Subsequent immunoblotting experiments confirmed and quantified this response, with an increase of phospho-CREB1 in excess of five-fold in PKCε-transfected cells when compared with the Ad0 control (*Figure 3B* and *C*).

### 3.3 CREB1 acts downstream of PKCε to regulate HO-1

Next, siRNA was used to interrogate the function of CREB1 in HO-1 induction downstream of PKCε. A single oligonucleotide and a pool of four distinct sequences depleted CREB1. The induction of HO-1 mRNA following expression of CA-PKCε was attenuated by CREB1 depletion, a response not seen in EC transfected with control siRNA (*Figure 3D*). Similarly, the up-regulation of HO-1 protein following activation of PKCε was inhibited by the CREB1 pooled oligonucleotides, with a less marked and more variable response seen with the single oligonucleotide (*Figure 3E*). These data indicate that CREB1 is a key component in PKCε-mediated HO-1 regulation.

### 3.4 PKCε, Nrf2, and the regulation of HO-1

**Figure 4:**
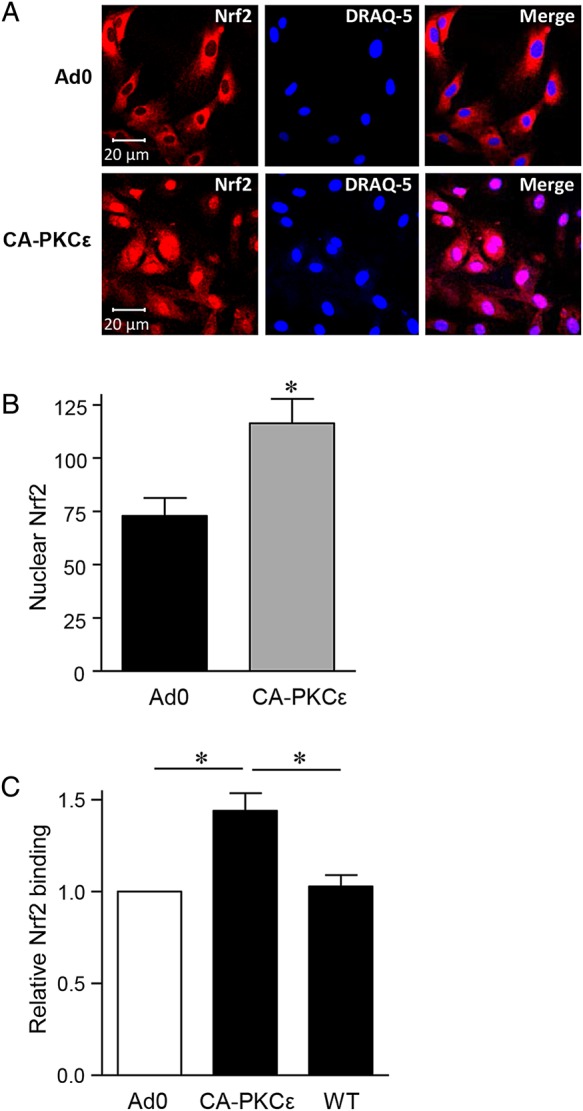
PKCε activation results in Nrf2 nuclear translocation. (*A*) HUVECs were transfected with CA-PKCε or Ad0 (MOI 100) for 16 h prior to staining with an anti-Nrf2 antibody and DRAQ-5 nuclear stain. Representative images are shown, and in (*B*) a histogram of pooled nuclear fluorescence data from five individual experiments, expressed as MFI of Nrf2 (mean ± SEM). (*C*) HUVECs were left UT or transfected with CA-PKCε or Ad0 (MOI 100) for 16 h. Nuclear lysates were generated and Nrf2 activation assessed using a DNA-binding ELISA in the presence and absence of a competitive WT oligonucleotide (WT), corresponding to the consensus sequence for Nrf2 binding. Pooled data (mean ± SEM) from at least four independent experiments are shown, corrected for tubulin expression where appropriate, and normalized to Ad0-transfected control cells (*t*-test, *P < 0.05).

**Figure 5:**
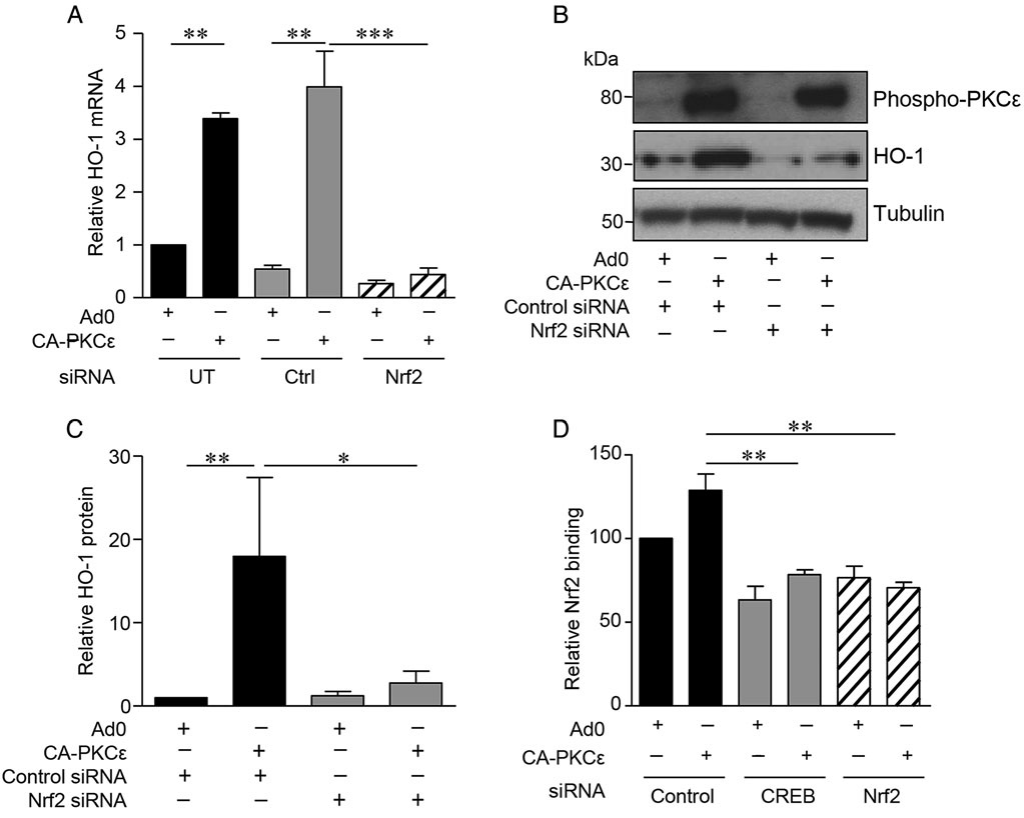
A PKCε-CREB-Nrf2 signalling pathway regulates HO-1 expression in EC. (*A*) HUVECs were left UT or transfected with pooled Nrf2 or Ctrl siRNA oligonucleotides (40 nM) for 24 h prior to transfection with Ad0 or CA-PKCε for 16 h. HO-1 mRNA levels were analysed by qRT-PCR. Pooled data are shown from three individual experiments and normalized to expression in EC transfected with Ad0 alone. (*B* and *C*) HUVECs were transfected with Nrf2 or control siRNA as above, prior to transfection with Ad0 or CA-PKCε for 24 h. HO-1 and phospho-PKCε^(Ser729)^ were analysed by immunoblotting. A representative immunoblot is shown (*B*), and the histogram (*C*) shows data from four individual experiments, corrected for tubulin expression, and normalized to control-transfected cells. (*D*) Nuclear lysates were obtained from HUVECs pretreated with control, Nrf2, or CREB siRNA oligonucleotides for 24 h prior to 16 h transfection with Ad0 or CA-PKCε. Nrf2 translocation was assessed using a DNA-binding ELISA. Pooled data from four independent experiments are shown (mean ± SEM) and normalized relative to Ad0-transfected control cells (ANOVA, *P < 0.05, **P < 0.01, ***P < 0.001).

The importance of the transcription factor Nrf2 in HO-1 regulation led us to investigate its potential role as a PKCε target in EC. Expression of CA-PKCε in HUVEC induced Nrf2 nuclear translocation, a response that was not seen with the Ad0 control (*Figure 4A* and *B*). A DNA-binding ELISA further demonstrated Nrf2 activation. A significant increase in Nrf2 DNA binding above that seen following transfection of the Ad0 control virus was observed following expression of CA-PKCε (*Figure 4C*). Moreover, this response was suppressed by inclusion of a competitive oligonucleotide corresponding to the consensus sequence for Nrf2 binding. Next, a pool of four distinct siRNA oligonucleotides was used to deplete Nrf2 (see Supplementary material online, *Figure S2D*), and this abrogated the PKCε-dependent increase in HO-1 mRNA (*Figure 5A*). Further investigation suggested that Nrf2 is a downstream target of activated PKCε. Nrf2 depletion failed to prevent PKCε phosphorylation in CA-PKCε-transfected HUVEC, while it attenuated the CA-PKCε-mediated increase in HO-1 protein (*Figure 5B* and *C*). Additional Nrf2 DNA-binding experiments suggested that PKCε activates Nrf2 via CREB1. Specifically, depletion of CREB1 with siRNA prevented the PKCε-mediated increase in Nrf2 DNA binding, a response reproduced as expected by Nrf2 siRNA (*Figure 5D*).

### 3.5 PKCε exerts cytoprotective and anti-inflammatory effects via induction of HO-1

**Figure 6:**
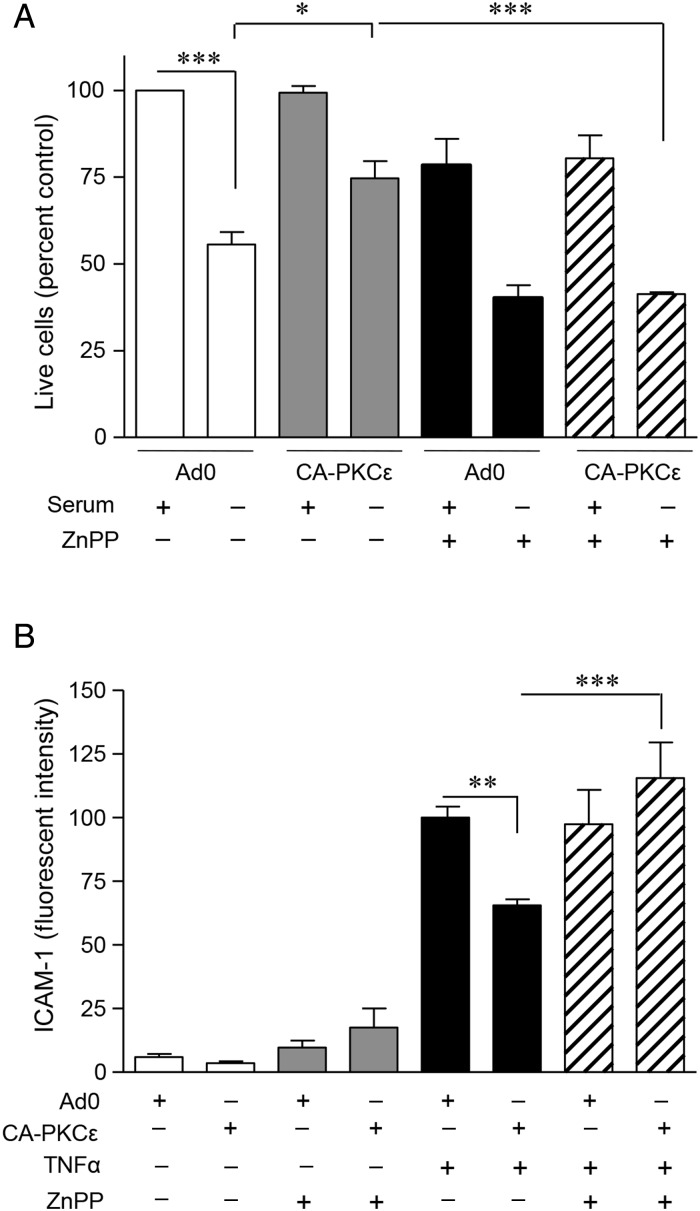
PKCε-induced HO-1 is cytoprotective and anti-inflammatory. (*A*) HUVECs seeded onto 24-well plates were transfected with CA-PKCε or Ad0 (MOI 100) for 24 h prior to culture in M199/10% FBS or in serum-depleted medium (M199/0.1% FBS), and in the presence or absence of the competitive HO inhibitor ZnPPIX (20 μM) for 24 h. ECs were incubated with MTS reagent in M199/5% FBS for 3 h and samples analysed on a plate reader at 490 nm. Pooled data from three independent experiments are shown. Change in the cell number is expressed as a percentage of the Ad0 control cultured in M199/10% FBS. (*B*) HUVECs were transfected as above prior to challenge with TNF-α 1 ng/mL for 6 h ± ZnPPIX. ICAM-1 expression was quantified by flow cytometry. Pooled data from four individual experiments are shown; changes in ICAM-1 are expressed as MFI (mean ± SEM) (ANOVA, *P < 0.05, **P < 0.01, ***P < 0.001).

In an effort to determine the functional significance of PKCε-mediated HO-1 induction, two separate models were used. In the first, serum starvation of HUVEC for 24 h resulted in up to 50% EC death. Overexpression of CA-PKCε reduced this cell loss from 50 to 25%, and this protection was not seen in cells treated with the Ad0 control (*Figure 6A*). The role of increased HO-1 activity in the protection afforded by PKCε was supported by its reversal in the presence of HO antagonist ZnPPIX (*Figure 6A*).

HO-1 also exerts important anti-inflammatory actions in the vascular endothelium.^5,25^ TNFα-mediated induction of ICAM-1 was used as a model response to investigate the role of HO-1 in PKCε-mediated anti-inflammatory effects. Adenoviral transfection of unstimulated EC in the presence or absence of ZNPPIX led to a modest non-significant effect on ICAM-1 surface expression. Treatment of EC with TNF-α resulted in a marked increase in cell surface ICAM-1, a response that was significantly attenuated by expression of CA-PKCε and not by the Ad0 control virus (*Figure 6B*). Moreover, although ZnPPIX had no effect on the induction of ICAM-1 by TNF-α, it reversed the anti-inflammatory action of PKCε, again demonstrating the importance of HO-1 as a PKCε target gene.

## 4. Discussion

PKCε activation is emerging as an important contributor to the maintenance of vascular EC homeostasis, through its ability to regulate anti-apoptotic and anti-inflammatory genes.^15,16^ A role for PKCε in VEGF-induced activation of Akt, Bcl-2, and eNOS, and in the protection of EC against serum starvation via modulation of eNOS activity, has also been reported.^16,26,27^ Moreover, when exposed to chronic hypoxia PKCε-deficient mice exhibit endothelial dysfunction, with reduced eNOS induction in pulmonary EC and increased vascular tone. This leads to pulmonary hypertension, which can be reversed by inhalation of NO.^28^ However, the precise role of PKCε-regulated signalling in endothelial homeostasis, and its potential as a therapeutic target remains to be determined. In the current study, we have identified a novel EC PKCε-CREB-Nrf2 signalling pathway that up-regulates expression of the vasculoprotective enzyme HO-1 and confers cytoprotection. In light of the severe vascular endothelial injury and accelerated atherosclerosis reported in *HMOX1* deficiency,^6,7^ we believe that this pathway is worthy of further study as an important contributor to endothelial homeostasis and resistance to endothelial injury.

A gain- and loss-of-function approach revealed a close relationship between PKCε activity and HO-1 expression. Selective activation of PKCε using an adenoviral vector increased HO-1, whereas HO-1 protein was depleted in EC and aortas from PKCε-deficient mice, when compared with WT littermates. Physiological agonists of PKCε have not been extensively studied in the vascular endothelium. We chose to investigate Ang II, an important vascular mediator which activates PKCε in cardiomyocytes^23^ and up-regulates HO-1 in EC.^29,30^ In addition to its role as a vasoconstrictor, Ang II activates intracellular signalling pathways to modulate the release of growth factors and cytokines, and to influence cell growth and proliferation.^31^ We propose that HO-1 induction by Ang II is part of a homeostatic response to minimize potentially deleterious cardiovascular effects of Ang II, with PKCε acting as an important intermediary. Treatment of EC with Ang II resulted in PKCε phosphorylation and HO-1 induction, a response inhibited by DN-PKCε. In addition to regulating oxidative stress, increased HO-1 activity is anti-inflammatory, attenuating Ang II-induced PGE2 and F2α synthesis.^32^ VEGF also activates PKCε in HUVEC and we have reported that DN-PKCε prevents VEGF-mediated up-regulation of the anti-apoptotic protein A1.^15^ However, inhibition of PKCε did not attenuate HO-1 induction by VEGF (not shown), suggesting that additional PKC isoforms may be involved,^22^ or that PKCε activity is not predominant for HO-1 regulation by VEGF and acts as a parallel pathway.

We have reported that inhibition of MEK-1, the upstream kinase of ERK1/2, attenuates PKCε-mediated induction of A1, A20, and Bcl-2,^15^ whereas the PI-3K antagonist LY290042 prevents celecoxib induction of HO-1.^12^ However, pharmacological antagonists of both pathways failed to prevent HO-1 up-regulation following PKCε activation. This led us to use a phosphokinase Ab array to identify alternative PKCε targets, and predominant among these was phosphorylation of CREB1. Although JNK and p38 MAPK have been associated with HO-1 regulation,^10,13^ no activation of p38 was seen in the antibody array and we have reported in EC that PKCε activity inhibits JNK phosphorylation.^15^

CREB1, which belongs to the CREB/ATF family of transcription factors, is predominantly located in the nucleus bound to CRE and is activated by phosphorylation at Ser^133^ in the kinase-inducible domain. CREB is universally expressed with roles in cell metabolism, differentiation and survival, and in cell-specific responses to extracellular stimuli.^33^ We found CREB1 to be constitutively expressed in human EC, and its phosphorylation was increased up to six-fold following PKCε activation. Moreover, depletion of CREB1 attenuated PKCε-mediated induction of HO-1. In human breast cancer cell lines, a link between PKCε and CREB1 was suggested by the observation that PKCε siRNA reduces constitutive CREB1^(Ser133)^ phosphorylation and Bcl-2 expression.^34^ Although the role of CREB1 in the vasculature remains to be fully understood, it appears to be protective, as arterial disease in rodent models of atherosclerosis, hypertension, and insulin resistance is closely associated with loss of aortic CREB1.^35^ Moreover, targeted cardiac expression of DN CREB1 enhances oxidative stress, mitochondrial dysfunction, apoptosis, and mortality in a murine model.^36^ These data suggest that CREB1 is important for the regulation of diverse protective genes. Indeed, in the vascular endothelium, CREB1 plays a central role in VEGF-driven angiogenesis, maintenance of endothelial barrier function, and in prostacyclin-induced cytoprotection,^37,38^ with both VEGF and prostacyclin able to induce HO-1.^39,40^

The *HMOX1* promoter, which comprises >10 kb, has binding sites for a variety of transcription factors that regulate basal and induced expression. In addition, the E1 and E2 upstream enhancer regions include ARE.^10^ Nrf2, a member of the Cap'n'Collar family of basic leucine zipper (bZip) transcription factors, binds to and drives ARE-mediated induction of HO-1 and other phase II anti-oxidant enzymes.^41^ Expression of CA-PKCε in human EC led to specific nuclear translocation of Nrf2, and increased binding of Nrf2 in nuclear lysates to an oligo containing an ARE consensus site. Furthermore, siRNA depletion of Nrf2 abrogated PKCε-induced HO-1 mRNA and protein up-regulation, whereas knockdown of either CREB1 or Nrf2 was able to attenuate Nrf2 binding to the ARE consensus oligonucleotide. Although this suggests that PKCε-CREB1-Nrf2 acts in a linear pathway with transcriptional co-operation between CREB1 and Nrf2, further studies are required to understand the precise molecular relationship between CREB1 and Nrf2.

CREB and Nrf2 are both members of the bZip family in which heterodimerization is common. Indeed, heterodimerization between Nrf2 and ATF4 is involved in the induction of HO-1 by cadmium chloride.^42^ Thus, a direct physical interaction may occur between Nrf2 and CREB1 resulting in co-operation at the *HMOX1* promoter. This is of particular relevance in light of the demonstration that CREB1 is directly involved in HO-1 transcription induced by oxidized phospholipids, via signalling pathways including PKC.^43^ However, such interactions may be cell type-specific with a complex network reported in dendritic cells in which activity of HO-1 and Nrf2 inhibits CREB1/ATF1 activation.^44^ In addition, phosphorylation of Nrf2 by PKC might be important. However, this interaction remains to be fully understood and may be both isoform and cell type-specific. Although PKC-mediated phosphorylation of Nrf2 at Serine 40 has been linked to HO-1 induction in cancer cell lines, PKCδ seems to be the isoform involved.^45^ Moreover, at least in hepatoblastoma cells, PKCε may phosphorylate Keap1 (INrf2) and so target Nrf2 for degradation. Depletion of PKCε in these cells, or decreased expression in human lung or liver tumours, was associated with increased expression of Nrf2 cytoprotective genes and enhanced cell survival.^46^ In contrast to these findings, other studies have reported that increased PKCε expression in breast and lung carcinomas correlates positively with pro-survival genes and aggressive metastatic behaviour.^47,48^ This relationship between PKCε activity and pro-survival genes more closely mirrors that found in the endothelium.^15,16,26^

The induction of HO-1 led us to explore its contribution to the protective actions of PKCε. Expression of CA-PKCε in EC attenuated both TNFα-mediated up-regulation of ICAM-1 and apoptosis induced by serum starvation. ZnPPIX, a pharmacological antagonist of HO activity, inhibited at least partially the cytoprotection afforded by PKCε activation. These data suggest that HO-1 is one of a number of PKCε-inducible protective genes, with others known to include the anti-apoptotic genes A1 and Bcl-2, and the anti-inflammatory A20 and eNOS.^15,16,26^ The regulation of A1, A20, and Bcl-2 by PKCε utilizes a distinct downstream ERK1/2-NF-κB-dependent pathway, while the anti-inflammatory effect of PKCε activity is additionally strengthened by its inhibition of TNFα-induced JNK phosphorylation.^15^

Current evidence demonstrates that PKCε is able to enhance vascular endothelial resistance to injury, via distinct signalling pathways including those regulated by ERK1/2, PI-3K, Akt, and NF-κB, and mediated by an array of downstream target genes. To these we have added induction of HO-1 via a CREB1 and Nrf2-dependent pathway. Given the potent cytoprotective actions of HO-1 and its products, this observation significantly extends the importance of PKCε activity in the maintenance of endothelial homeostasis and resistance to injury. The findings raise the possibility that once completely defined, PKCε signalling pathways in the vascular endothelium may represent a novel target through which the vascular endothelium can be therapeutically conditioned.

## Supplementary material

Supplementary material is available at *Cardiovascular Research* online.

**Conflict of interest:** none declared.

## Funding

This work was supported by Arthritis Research UK (grant number 18252). The authors also acknowledge support from the National Institute for Health Research Biomedical Research Centre based at Imperial College Healthcare NHS Trust and Imperial College London. Funding to pay the Open Access publication charges for this article was provided by the Imperial College London Open Access funding scheme.
